# Supplementary material for: Senescent cells enhance newt limb regeneration by promoting muscle dedifferentiation
Source: Aging Cell. 2023 Apr 6;22(6):e13826. doi: 10.1111/acel.13826 (PMC10265169; doi:10.1111/acel.13826)
Supplement: Supplementary file 1 — Appendix S1. [file ACEL-22-e13826-s002.docx]

**Supplementary Material**

**Title:** Senescent cells enhance newt limb regeneration by promoting muscle dedifferentiation

**Running title:** Senescence enhances dedifferentiation **Authors:** Hannah E. Walters^1*^, Konstantin E. Troyanovskiy^1,2^, Alwin M. Graf^1^ and Maximina H. Yun^1,3,4*^

**Supplementary Figures S1 to S10**

**Tables S1 to S2**

**Description of additional Supplementary Data S1 to S4**


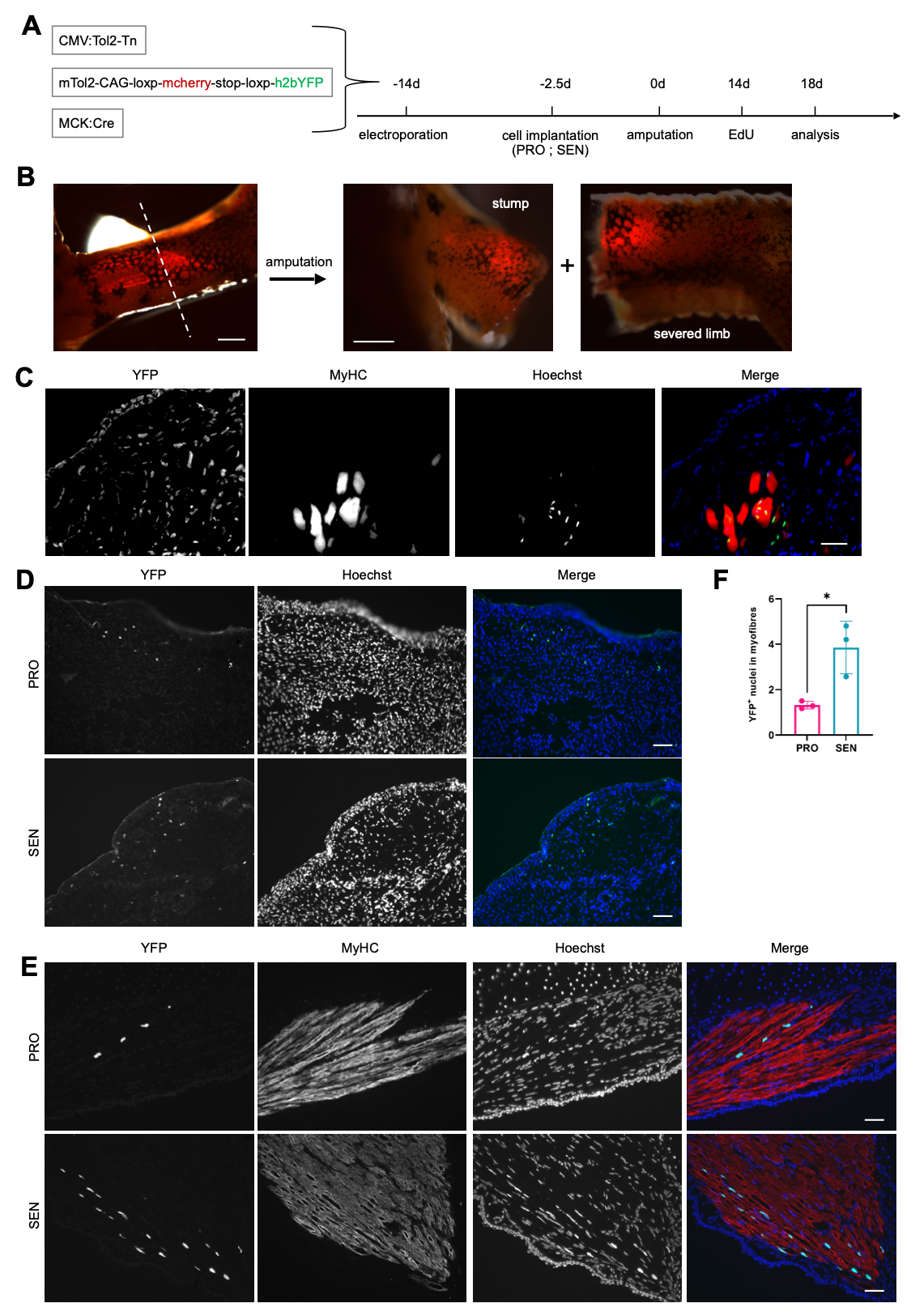


**Supplementary Figure 1. Genetic strategy for labelling myofibres and fate-tracing dedifferentiated progenitors *in vivo*.**

**(A)** Schematic depicting *in vivo* myofibre labelling approach to enable fate-tracing of muscle-derived dedifferentiated progenitor cells during regeneration.

**(B)** Representative image of a newt limb following electroporation with the indicated constructs and cell implantation (left, at 0d). Limb amputation, performed through the centre of the electroporated area (yellow dotted line), results in a stump (middle) which contains genetically labelled myofibres. Scale bar 500 μm.

**(C)** Representative immunofluorescence image of a stump cryosection depicting YFP^+^/MyHC^+^ myofibres, as detected through α-GFP (green, iii) and α-MyHC (red, ii) antibody staining, with nuclear counterstaining shown in blue (i). Their progeny can be subsequently traced during limb regeneration through the expression of nuclear YFP. Scale bar 50 μm.

**(D)** Representative immunofluorescence images of YFP^+^ progenitors from proliferating control or senescent cell implanted limbs, as detected through α-GFP (green) antibody staining, with nuclear counterstaining shown in blue.

**(E)** Representative immunofluorescence images of regenerated limb sections showing re-differentiated YFP^+^ nuclei within muscle tissue (using α-GFP (green) and α-MyHC (red) antibody staining) from limbs implanted with proliferating control or senescent cell prior to amputation, with nuclear counterstaining shown in blue.

**(F)** Quantification of the percentage of YFP^+^ progenitors contributing to MyHC^+^ differentiated muscle fibres in regenerated limbs, following proliferating or senescent cell implantation (staining as in (E)). Two-tailed unpaired student’s t-test, *: p<0.05.

**
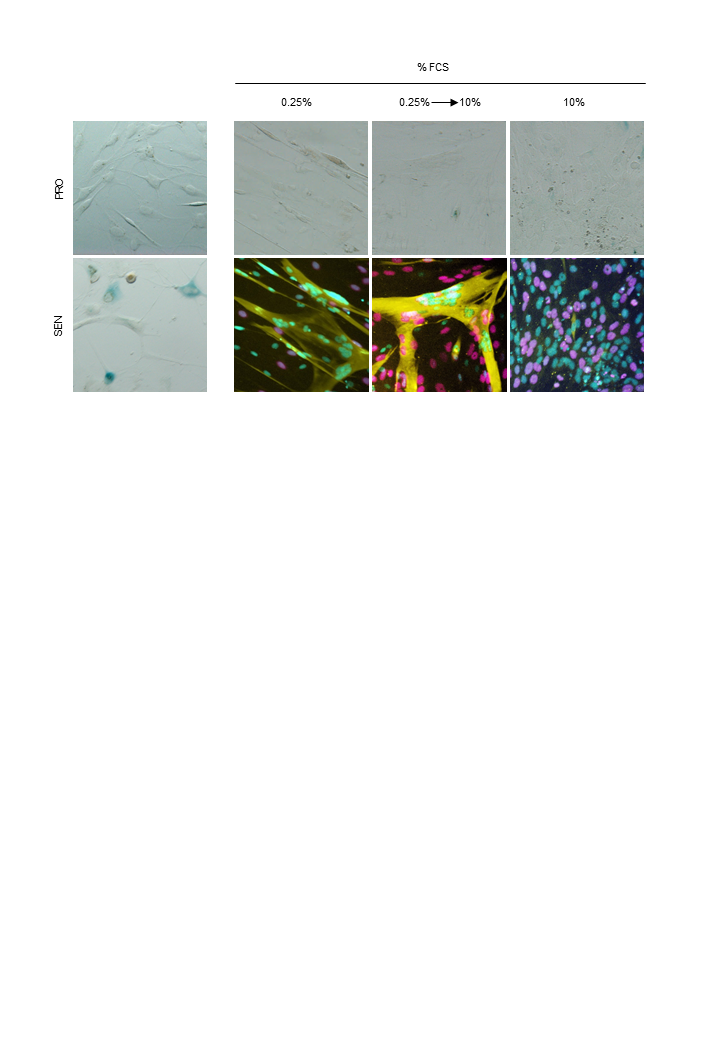
**

**Supplementary Figure 2. Senescence is not induced by cell-cell myogenic fusion or dedifferentiation events.**

Top row: Representative images of differentiated (0.25% FCS), dedifferentiated (0.25% 🡪 10% FCS) or undifferentiated A1 cultures (10% FCS) after SA-β-gal staining (blue in brightfield). Control senescent (‘SEN’) and untreated proliferating (‘PRO’) cultures were used as positive and negative controls respectively.

Bottom row: Representative images of the indicated cultures after staining against α-MyHC (yellow), EdU (magenta) and Hoechst (cyan). Note EdU incorporation in dedifferentiating myotube nuclei (magenta). n=4.

**
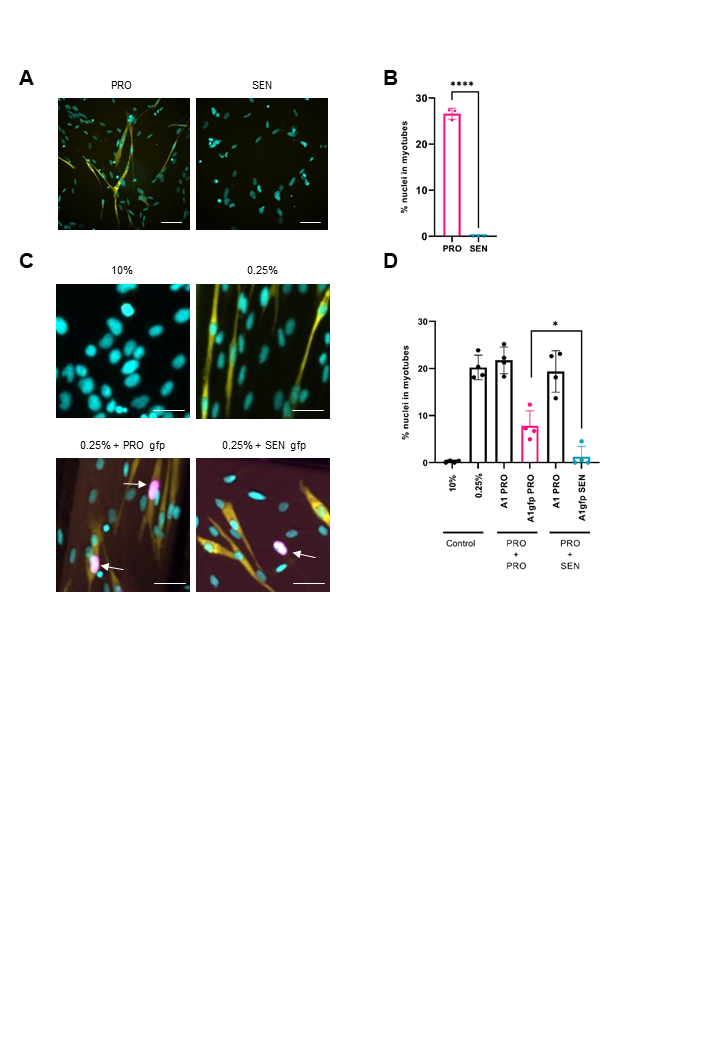
**

**Supplementary Figure 3. Senescence induction ablates myogenic potential.**

**(A, B)** Representative images of senescent (SEN) and control proliferating (PRO) cells, 5 days after treatment with differentiation media (0.25% FCS) and subsequent immunostaining (Hoechst; cyan, α-MyHC; yellow). The proportion of nuclei present in myotubes was quantified **(B)** from n>50 nuclei per replicate (n=3).

**(C)** Representative images of cultured cells following immunostaining against MyHC (yellow) and GFP (magenta), and counterstaining with Hoechst (cyan). A1n*gfp* senescent (‘SEN gfp’) and control proliferating (‘PRO gfp’) cells were generated *in vitro* and subsequently lifted and seeded into co-culture with A1 cells at high confluence. Co-cultures were then treated with differentiation media for 5 days before fixation and staining. White arrowheads indicate GFP^+^ nuclei.

**(D)** Percentage of nuclei present in myotubes (n>50 nuclei per replicate, n=3).

Two-tailed unpaired student’s t-tests were used to compare myogenesis between proliferating and senescent cells in both experimental set-ups (*: p<0.05, ****: p<0.0001).


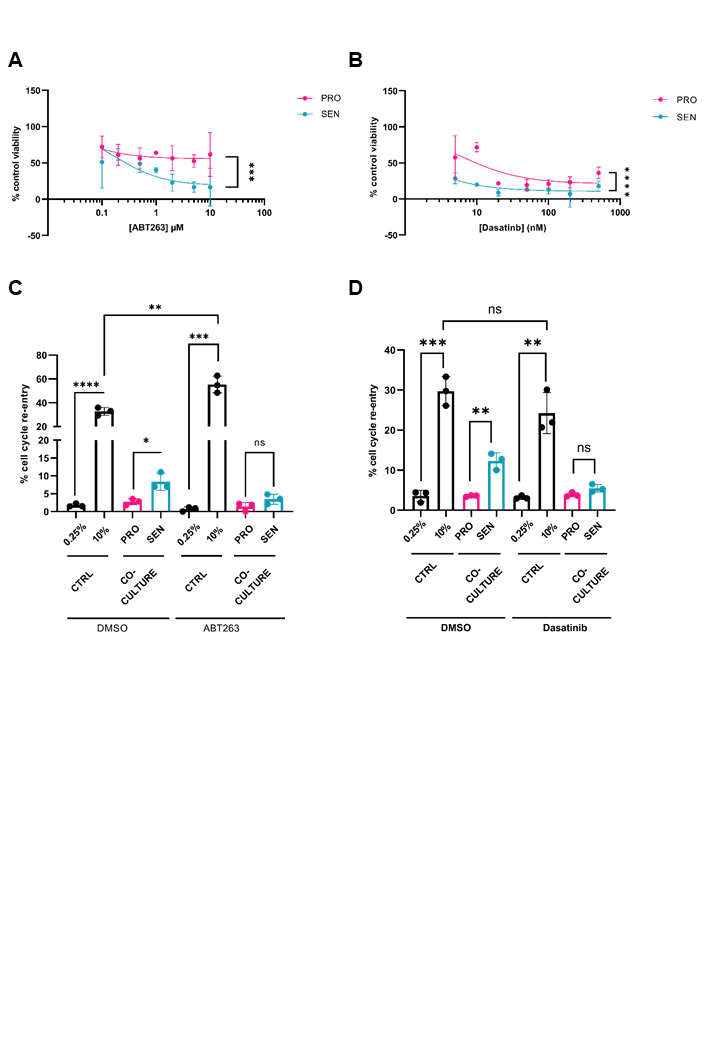


**Supplementary Figure 4. Pro-cell cycle re-entry effects of senescent cells are abrogated by senolytic treatment**

**(A,B)** Proliferating or senescent A1 cells (in 10% FCS normal growth media) were exposed to a dose-range of ABT263 or dasatinib for 72 hours before quantification of viable cells by fluorimetric assessment, based on the cell metabolic indicator alamarBlue (n=3). Two-way ANOVA were used for statistical analysis.

**(C,D)** Quantification of the proportion of myotube nuclei undergoing cell cycle re-entry, 72h post-treatment with the indicated conditions (DMSO vehicle control, ABT263 at 1 μM and dasatinib at 10 nM). Cultures were treated in 0.25% or 10% FCS control media (CTRL), or in co-culture with proliferating or senescent cells, all in 0.25% FCS media. Cell cycle re-entry was quantified as the proportion of nuclei (stained using Hoechst) within myotubes (stained using α-MyHC) showing EdU incorporation. Two-tailed unpaired student’s t-tests were used to compare data in **(C)** (*: p<0.05, **: p<0.01, ***:p<0.001, ****: p<0.0001, ns: not significant).

**
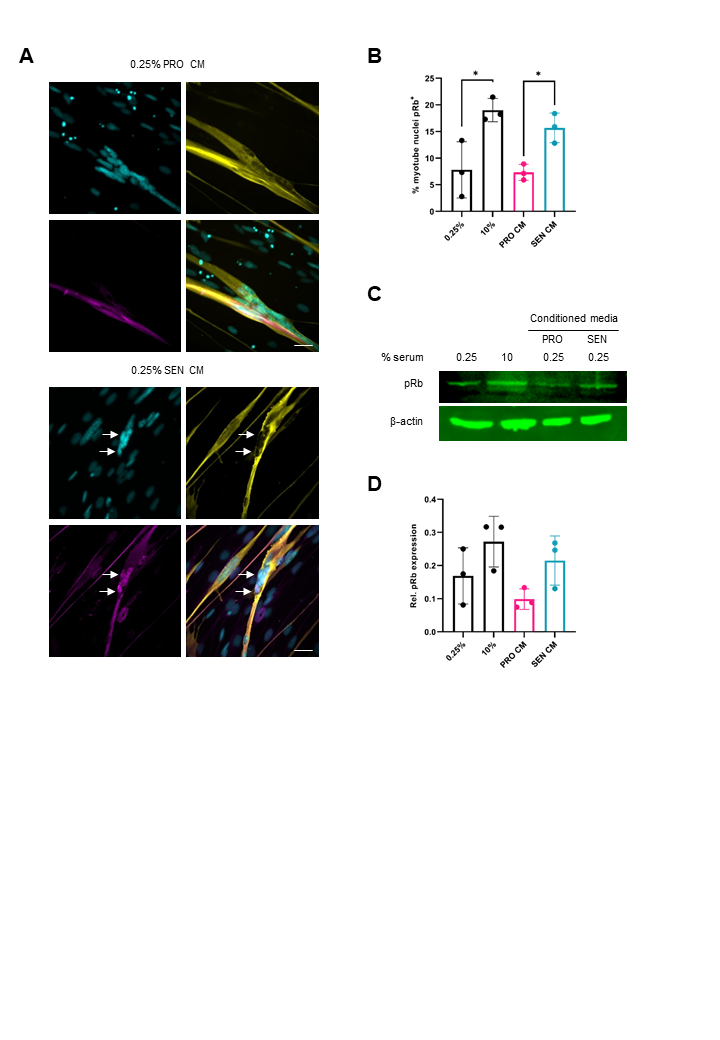
**

**Supplementary Figure 5: Senescence-induced cell cycle re-entry is accompanied by phosphorylation of Rb**

**(A)** Representative images of myotubes following immunostaining against MyHC (yellow), pRb (magenta) and Hoechst (cyan) labelling for the indicated conditions, 72hs post-treatment. White arrows indicate EdU^+^ nuclei within myotubes.

**(B)**: Quantification of the proportion of myotube nuclei displaying pRb, as in **(B).** Statistical analysis performed using two-tailed unpaired student’s t-test, *: p<0.05 (n=3).

**(C)**: Representative Western blot against pRb for the indicated conditions. β-actin was used as loading control.

**(D)**: Quantification of signal intensity. pRb band intensities were normalized against β-actin loading controls (n=3).


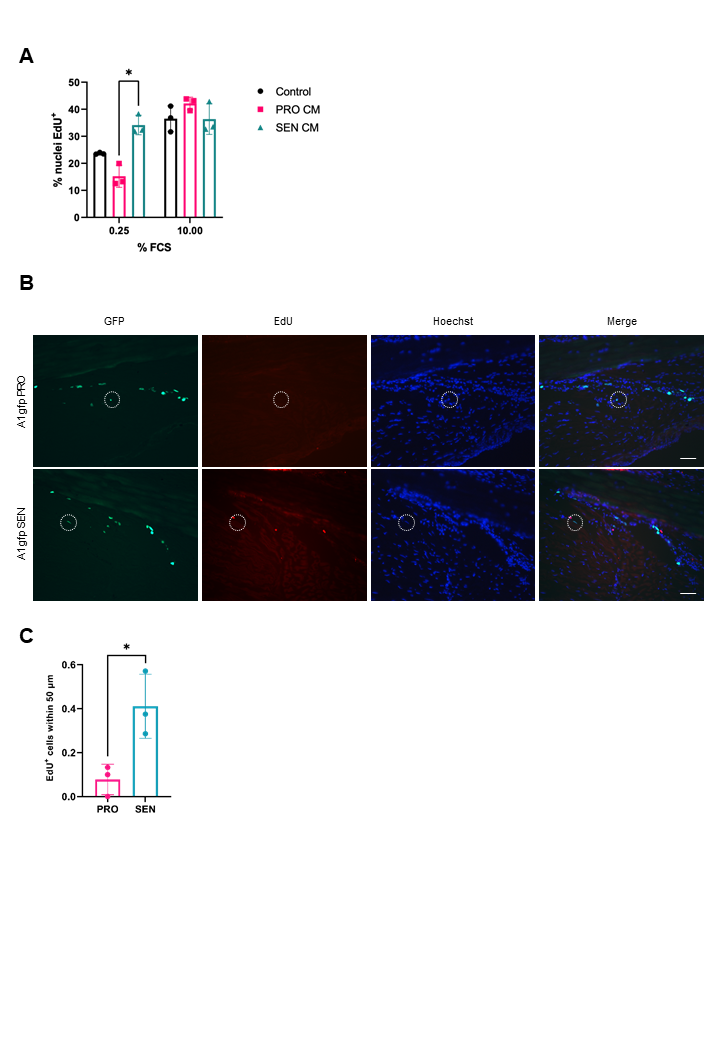


**Supplementary Figure 6: Senescence promotes cellular proliferation in a non-cell-autonomous manner**

**(A)** A1 cells were exposed to fresh or 48-hour conditioned media containing 0.25% or 10% FCS for 72 hours before proliferation was assessed, as the % nuclei showing EdU incorporation (n-3, representative data from one of two independent experiments shown). ANOVA and post-hoc Tukey tests were used for statistical analysis (*p<0.05).

**(B, C)** Control proliferating or senescent A1gfp cells were implanted into contralateral limb tissue. Local proliferation was assessed by EdU incorporation analysis, scoring the number of EdU^+^ nuclei in direct proximity (within a 50 μm radius, dashed circle) to implanted cells. Representative images shown in **(B)** (scale bar 100 μm), with quantification in **(C)** (*: p<0.05, paired student t-test).

**
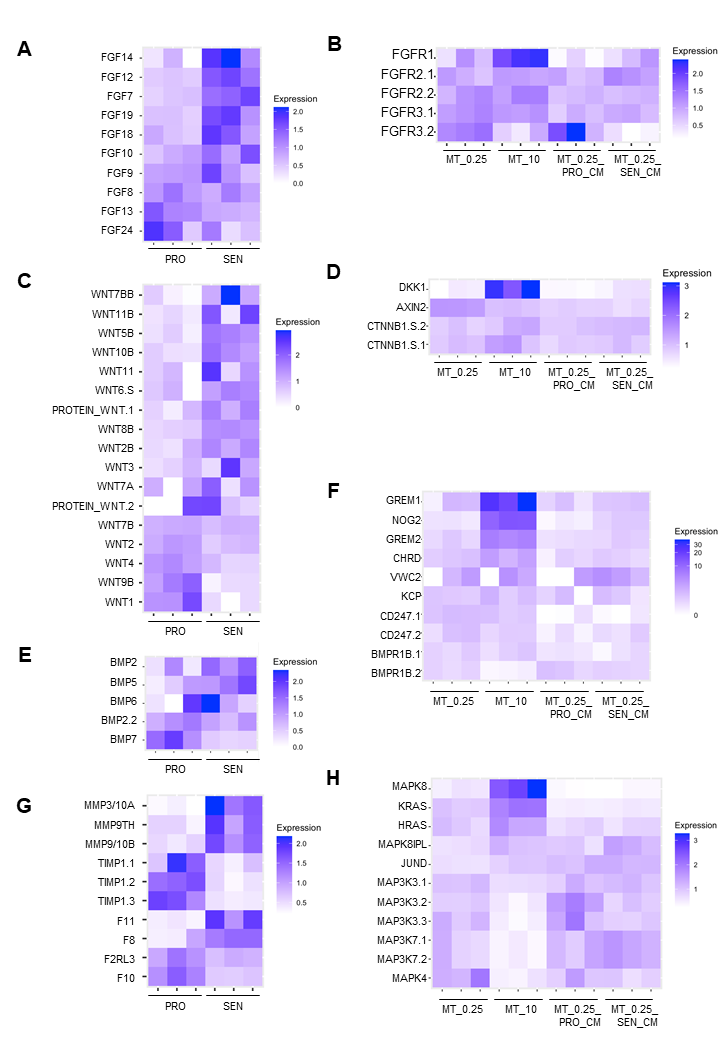
**

**Supplementary Figure 7. Candidate pathways involved in mediating senescence-induced dedifferentiation*.***

**(A-H)** heatmaps depicting transcriptional changes between proliferating vs. senescent cells **(A, C, E, G)** or between differentiated and dedifferentiated myotubes upon serum or senescent CM exposure **(B, D, F, H)**. Ligands and downstream signalling transcripts are depicted for the FGF pathway **(A, B)**, for Wnt signalling **(C, D)** for BMP signalling **(E, F)** and for protease expression **(G)** and ERK signalling **(H)**. Transcript expression for each replicate is normalized relative to the mean reads per million transcripts across all sample groups in each heatmap. Supplementary Data S3 contains tables with expression counts, log2fold change and adjusted p-values for each comparison.

**
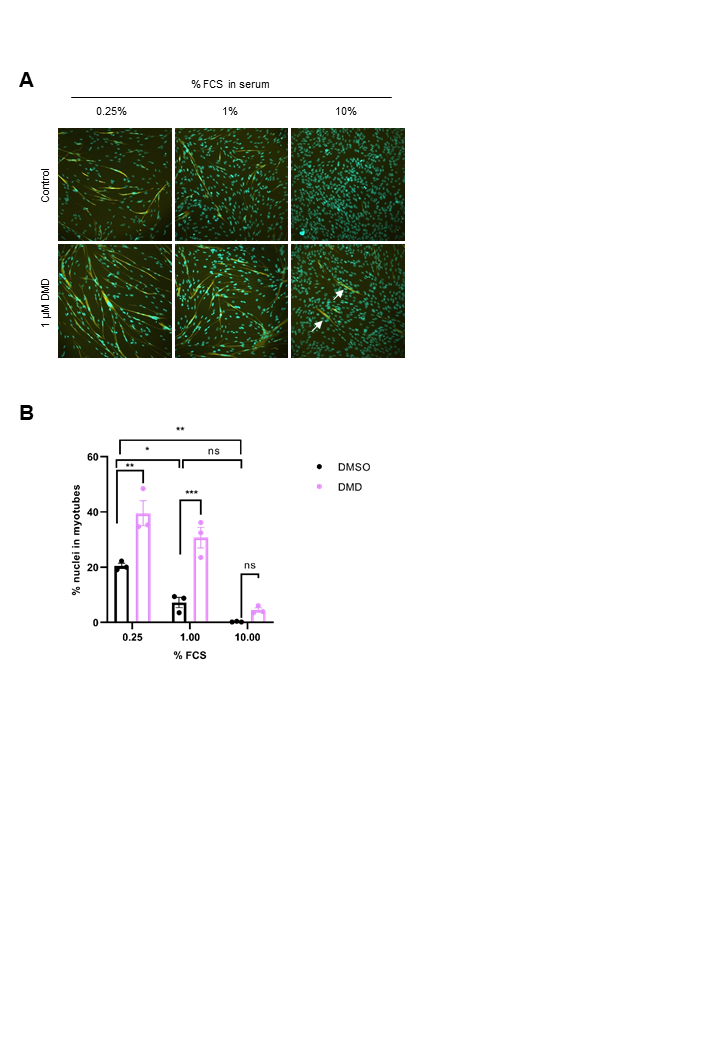
**

**Supplementary Figure 8. BMP inhibition promotes myogenesis.**

**(A, B)** A1 cells were seeded at high confluence and left to bed down before exposure to 1 μM dorsomorphin (DMD) or vehicle only (DMSO) in 0.25%, 1% or 10% FCS-supplemented media for 5 days. Cells were then fixed and immunostained with α-MyHC and counter-stained with Hoechst. Representative images shown in **(A)**, quantification of % nuclei in myotubes in **(B)**. White arrows indicate myotube formation in 10% serum upon DMD treatment. Two-way ANOVA and post-hoc Tukey tests were used for statistical analysis (*: p<0.05, **: p<0.01, ***: p<0.001, ns: not significant). Representative data shown from 2 independent experiments (n=3 technical replicates).


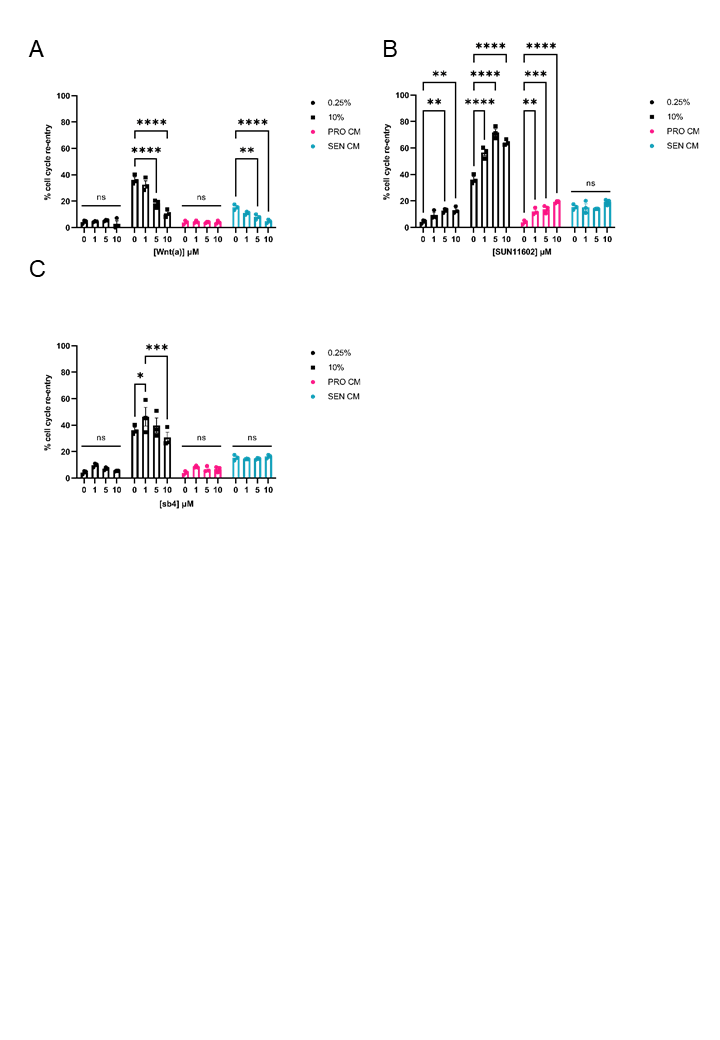


**Supplementary Figure 9. FGFR1 activation enhances cell cycle re-entry**

**(A-C)** Quantification of the proportion of myotubes undergoing cell cycle re-rentry for the indicated conditions, 72h post-treatment. Myotubes were generated and subsequently exposed to DMSO vehicle control or inhibitors in 10% **(A)** or 0.25% **(B)** FCS in the presence of proliferating/senescent conditioned media treatment. Inhibitors were used over a non-toxic dose range. Cell cycle re-entry was quantified as the proportion of myotube (MyHC^+^) nuclei showing EdU incorporation. Statistical analysis was performed using two way ANOVA and Tukey post-hoc multiple comparisons testing (*: p<0.05, **: p<0.01, ***: p<0.001, ****: p<0.0001, ns: not significant). Representative data from n>2 experiments shown.


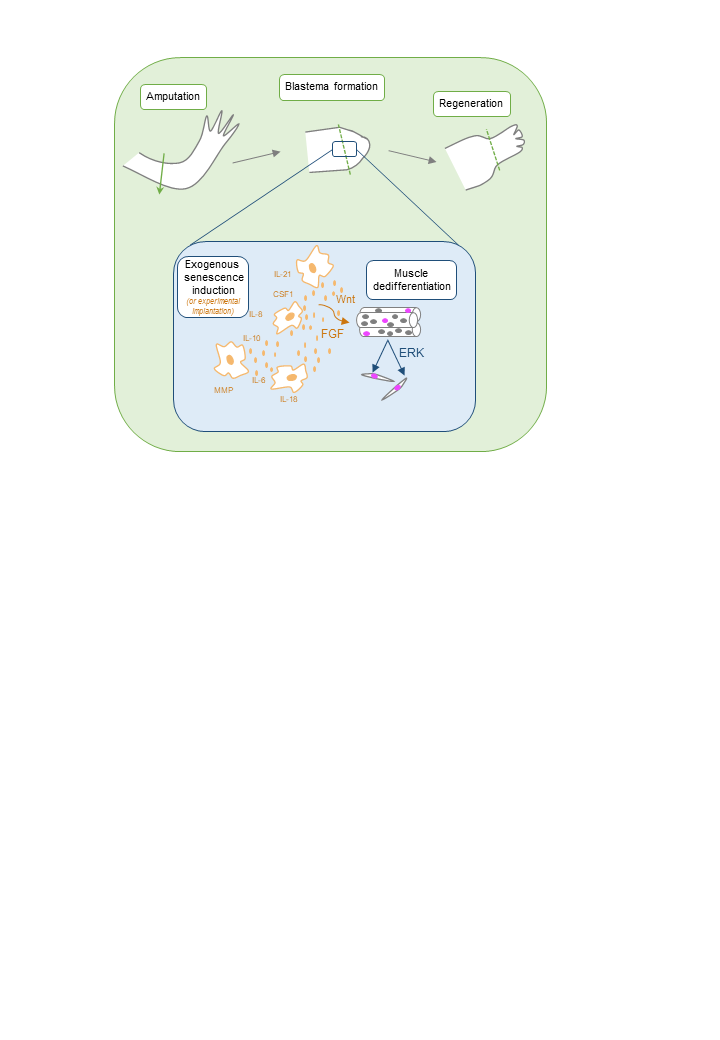


**Supplementary Figure 10. Proposed mechanism of promotion of dedifferentiation by senescent cells during newt limb regeneration.**

Schematic depiction of the proposed mechanism for how senescent cells enhance limb regeneration. Implantation of additional senescent cells (to expand the pool of senescent cells dynamically induced during regeneration) leads to enhanced muscle dedifferentiation, suggesting senescent cells promote dedifferentiation in a non-cell autonomous manner. Our *in vitro* data highlights the FGF-ERK signalling axis as a critical mediator which facilitates the generation of myogenic progenitors. Following many rounds of proliferation within the blastema, these progenitors subsequently redifferentiate (in a process possibly involving BMP inhibition), to accomplish muscle regeneration.

**Table S1. Inhibitors used in this study**

| **Inhibitor** | **Target** | **Dose used (μM)** |
| --- | --- | --- |
| ABT263 | BCL2 (senolytic) | 1 |
| Dasatinib | RTK (senolytic) | 0.01 |
| Dorsomorphin | BMP antagonist | 1 |
| U0126 | MEK1/2 (ERK) | 10 |
| PD173074 | FGFR1 | 1 |
| AZD4547 | FGFR1,2,3 | 5 |
| C59 | Wnt | 1 |
| AEBSF | Serine protease | 20 |
| GM6001 | Broad spectrum MMP | 2 |
| Wnt agonist | Wnt signalling | 1-10 |
| Sb4 | BMP4 mimetic | 1-10 |
| SUN11602 | FGFR1 agonist | 1-10 |
| Etoposide | Topoisomerase II | 20 |
| Nutlin-3a | p53/MDM2 | 1 |

**Table S2. Antibodies used in this study.**

| Target | Supplier and code | Application | Dilution |
| --- | --- | --- | --- |
| **Primary** |  |  |  |
| α -myosin heavy chain | Custom | Immunofluorescence | 1:1000 |
|  |  | Western blotting | 1:2000 |
| α-phospho-Rb | Cell signalling (9308) | Immunofluorescence | 1:200 |
|  |  | Western blotting | 1:500 |
| α-GFP | Abcam (ab6673) | Immunofluorescence | 1:1000 |
| **Secondary** |  |  |  |
| α-mouse 680/800  α -rabbit 680/800 | Licor | Western blotting | 1:2000 |
| Alexafluor α-mouse/rabbit 488/594/647 | Invitrogen | Immunofluorescence | 1:1000 |

**Other Supplementary Materials for this manuscript include the following:**

**Data S1. DGE analysis for all conditions described in Figure 3**

**Data S2. List of genes corresponding to the GO pathway enrichment analysis corresponding to Figure 3 and adjusted p-values.**

**Data S3. Expression counts, log2fold change and adjusted p-values for transcripts shown in RNAseq heatmaps in Figure 3 and Supplementary Figure 7.**

**Data S4. Statistical analysis corresponding to Figure 4.**
